# Avoiding Error and Finding the Right Balance in European Health Technology Assessments: Insights Generated by the European Access Academy

**DOI:** 10.3390/jmahp13010006

**Published:** 2025-02-10

**Authors:** Elaine Julian, Tom Belleman, Maria João Garcia, Maureen Rutten-van Mölken, Robin Doeswijk, Rosa Giuliani, Bernhard J. Wörmann, Daniel Widmer, Patrick Tilleul, Ruben Casado Arroyo, Valentina Strammiello, Kate Morgan, Marcus Guardian, Michael Ermisch, Renato Bernardini, Fabrizio Gianfrate, Stefano Capri, Carin A. Uyl-de Groot, Mira Pavlovic, Jörg Ruof

**Affiliations:** 1 Secretariat of the European Access Academy (EAA), 4059 Basel, Switzerland; 2 Erasmus School of Health Policy and Management, Department Health Technology Assessment, Erasmus University Rotterdam, 3062 PA Rotterdam, The Netherlandsm.rutten@eshpm.eur.nl (M.R.-v.M.);; 3 F. Hoffmann-La Roche Ltd., 4070 Basel, Switzerland; 4 Institute for Medical Technology Assessment, Erasmus University Rotterdam, 3062 PA Rotterdam, The Netherlands; 5 European Hematology Association (EHA), 2514 AA The Hague, The Netherlands; 6 Guy’s and St Thomas’ NHS Foundation Trust, London SE1 7EH, UK; 7 German Association of Hematology and Oncology (DGHO), 10117 Berlin, Germany; 8 Division of Hematology, Oncology and Tumor Immunology, Department of Medicine, Charité-Universitätsmedizin Berlin, 10117 Berlin, Germany; 9 European Union of General Practitioners (UEMO), 1000 Brussels, Belgium; 10 APHP and Faculty of Health, University of Paris-Cité (Former Member), 75006 Paris, France; 11 Cardiac Electrophysiology Laboratory, Université Libre de Bruxelles- Erasme Hospital, 1070 Brussels, Belgium; 12 European Society of Cardiology (ESC), 06903 Sophia Antipolis, France; 13 European Patients’ Forum (EPF), 1040 Etterbeek, Belgium; 14 Myeloma Patients Europe (MPE), 1050 Brussels, Belgium; 15 International Horizon Scanning Initiative (IHSI), 1140 Brussels, Belgium; 16 Medicines Division, National Association of Statutory Health Insurance Funds (GKV-SV), 10117 Berlin, Germany; 17 Department of Biomedical and Biotechnological Sciences (BIOMETEC), Section of Pharmacology, University of Catania, 95124 Catania, Italy; 18 Department of Health Economics, University of Ferrara, 44121 Ferrara, Italy; 19 School of Economics and Management, Cattaneo-LIUC University, 21053 Castellanza, Italy; 20 Medicines Development and Training (MDT) Services, 75020 Paris, France; 21 Sabouraud Research and Treatment Center for Scalp and Skin Diseases, 75010 Paris, France; 22 Hannover Medical School, Institute for Epidemiology, Social Medicine and Health System Research, 30625 Hannover, Germany

**Keywords:** EUHTA, health policy, best available evidence, health technology assessment, European medicines agency, European access academy

## Abstract

Background: We examined four potential challenges for the implementation of the European Union (EU) Regulation 2021/2282 on Health Technology Assessment (EU HTAR): interaction with the European Medicines Agency (EMA), expert input, the interface of European health technology assessment (EU HTA) joint procedures with those within Member States, and the management of conflict of interest. This research aims to explore how to address these challenges in a balanced manner and prioritise key actions for effective collaboration in the context of the EU HTA. Methods: The methodology included a pre-convention survey among relevant stakeholders as well as working groups and the plenary ranking of discussion outcomes at the European Access Academy (EAA) Spring Convention 2024. Results: In the survey, 65.5% of respondents indicated that experts are currently not sufficiently included in the upcoming joint scientific consultations and clinical assessments; only 37.9% suggested that the EU HTA joint procedures would accelerate national appraisal decision-making, and 58.6% believed that the principles of ‘transparency’ and ‘competency’ are balanced in the EU HTA position on conflict of interest. The top priority action points identified in the working groups were the involvement of the best available expertise, the early and inclusive involvement of experts, strengthened early scientific dialogue, and the fostering of the political willingness/financial support of EU Member States to increase capacities. Conclusions: The key topics identified were an approach to conflict of interest that balances transparency obligations and the need for expertise, strengthens the involvement of clinical and patient experts, intensifies early interaction between the EMA and EU HTA, and increases the involvement of the EU Member States.

## 1. Introduction

When applying the statistical concept of type I and type II errors to health technology assessment (HTA), there are two fundamental sources of error: (i) suggesting an additional benefit for a technology that does not offer better outcomes than available treatments or (ii) not acknowledging an additional benefit that does exist [1]. The first would lead to additional costs for health systems without providing improved treatment results for patients, while the latter would save health systems’ resources but withhold treatment options from patients who could benefit from them.

Finding the right balance between those two types of error is at the heart of any health technology assessment and is the ultimate aim of the European Union (EU) Regulation 2021/2282 on Health Technology Assessment (EU HTAR): ‘*HTA is able to contribute to the promotion of innovation, which offers the best outcomes for patients and society as a whole, and is an important tool for ensuring proper application and use of health technologies*’ [2]. Further, ‘*strengthening the balance in the pharmaceutical systems in the European Union and its Member States*’ is an important goal of the Commission and the Council [3].

In the joint European HTA process (EU HTA), a range of stakeholders and collaborators are involved and/or affected, whose interests, needs and requirements naturally differ. To achieve the goals of the regulation—improving the quality of HTA, reducing duplication, and ultimately to improving patient access to life-saving innovative health technologies—a careful balance across varying perspectives is essential. Approaching a balance means navigating the spectrum between being “too lenient” and “too strict” (i.e., minimizing type I and type II errors).

Several implementing regulations—or ‘implementing acts’ (IAs)—are currently being developed and are setting the framework and requirements for specific topics such as interaction with the European Medicines Agency (EMA), joint clinical assessments (JCA), and the management of conflict of interest (CoI) [4,5,6,7]. Those rules need to be carefully balanced to achieve the aims of the EU HTAR, and satisfy the needs of the Member States (MSs) and the stakeholders involved, in particular with respect to the following four challenges:

- Balancing Interaction: EU HTA and EMA [7].

The EU HTAR states that collaboration and alignment with the EMA is required for effective information exchange and procedural synchronisation [8]. Such collaboration is an important factor in facilitating efficient and predictable JCAs for all stakeholders concerned [9,10,11,12]. For this, it is important that data included in the EMA’s benefit-risk assessment and in the comparative JCA are overall aligned, while respecting the different remits of the two procedures. In addition, the JCA’s procedural timelines as defined in the respective IA are intended to be consistent with the EMA procedure [5,8].

- Balancing Expert Input: Joint Scientific Consultation (JSC) and JCA [5].

To ensure that joint HTA work is of high scientific quality and assesses, in accordance with the current standard of care, experts—both patients and clinicians—are offered participation in the joint work, as per the regulation [5,6,8]. Clinicians provide their specific expertise on the clinical context to inform the JCA and JSC [9,12,13,14], and patients offer their experiences to contextualise HTA [12]. To allow for this crucial input to be included, clinicians and patients will be required to obtain knowledge regarding HTA in general and the specific questions to be answered by HTA, as addressed in various projects of the European Commission [15,16]. In addition, processes for expert involvement need to be clear and efficient but have so far only been defined by a preliminary guidance developed by the joint consortium of 13 HTA agencies across the EU called EUnetHTA 21 [17]. Hence, the main challenge for expert input is balancing these requirements with the evident need for including their expertise and experience.

- Balancing Interface: EU HTA and MS (clinician and patient perspective) [5]

An additional layer of complexity for expert input arises from challenges regarding interaction between EU HTA-level and national-level societies and associations. While some clinical or scientific societies are well aligned with diagnostic and treatment guidelines at the EU level, others are rather fragmented and respective national guidelines differ (14,18). The same applies to the interaction levels of patient associations. Clear and aligned evaluation processes both at the EU and national level are required to reduce fragmentation, the duplication of work and to strengthen the validity and impact of their input.

- Balancing Bias due to Stakeholder Interests: Transparency and Expertise [6]

The EU HTAR states that experts involved in the joint procedures, selected for their expertise, act in individual capacity rather than representing an organisation, institution, or MS. To ensure the independent, impartial, and transparent participation of these experts, the EU HTAR and the IA on CoI require that only experts with no CoI, that is, those serving in an individual capacity with no financial or other stake, participate in the joint work [5,6,8]. However, given the foreseeable limited capacity of qualified experts, this can probably not be enforced too strictly. A balanced perspective on the two guiding principles of ‘transparency about individual interests’ and ‘expertise’ is required to guarantee appropriate access to relevant expertise throughout the process.

The 2024 Spring Convention of the European Access Academy (EAA), focused on ’EU HTA—Finding the Right Balance’, aimed at identifying an optimal balance in those controversial elements of the EU HTA framework. While specific challenges for the implementation of the EU HTAR have previously been discussed by the EAA and others, a more holistic view on balancing the various important perspectives is so far lacking [18,19,20,21,22]. The convention placed a special emphasis on interaction and cooperation within those four focus topics. The goal of this research is to investigate how such balance can be achieved and how key action points have to be prioritised for future effective and meaningful interaction and collaboration among involved parties in the EU HTA system.

## 2. Methods

The approaches to the pre-convention survey design, validation, finalisation, and analysis, as well as preparation, procedures, and data handling and analysis for the EAA Spring Convention 2024 were consistent with past EAA practice [14]. Details, where specific to the approach for the current work, are described below.

### 2.1. The Generation of Input for Discussions Through a Pre-Convention Survey

In preparation for the EAA Spring Convention 2024, the EAA Faculty created, distributed, and assessed an online survey (see Supplementary Materials) to gather insights into the perspectives of various stakeholders on finding the optimal balance on key topics relating to the EU HTA, with a focus on interaction with other individuals/parties. The semi-quantitative online survey included rating questions, ranking questions, as well as qualitative free-text questions.

For the rating questions an ordinal Likert Response Scale (with the options “yes”, “rather yes”, “rather no”, “no”) was utilised. As Likert scales are typically used for measuring degrees of agreement, satisfaction, and nuanced attitudes, ideal for capturing opinions, perceptions, and preferences, this tool was deemed most appropriate in this context where uncertainties regarding the implementation of the EU HTAR and therefore regarding the questions asked in this survey prevail [23].

A modified Delphi procedure was employed to develop the questionnaire as described previously [14,24]. The review panel included experts from several European countries (Belgium, France, Germany, Italy, Portugal, Spain, Switzerland, United Kingdom, and The Netherlands) and various institutional backgrounds and expertise, including clinicians, patients’ representatives, regulators, HTA bodies, payers, academia, and health technology developers (HTDs).

Between 18 January 2024 and 20 April 2024, the finalised survey was distributed through various channels, including LinkedIn, X (formerly Twitter), direct e-mail to the EAA network, and platforms such as the EAA website and the EU Health Policy Platform, to gather insights from key stakeholders and collaborators as previously defined [13,14].

Data analysis was performed as published earlier [14]. A preliminary analysis of responses was carried out for presentation at the EAA Spring Convention 2024 and publication in the Convention Proceedings [1]. Following the final data cut (20 April 2024), the quantitative response items were subjected to predetermined descriptive analyses. Complete pseudonymised survey data and free-text responses are available upon reasonable request.

### 2.2. Preparation of Break-Out Sessions During the EAA Convention

The EAA’s 2024 Spring Convention was held on April 18/19 at the Erasmus School of Health Policy and Management, Erasmus University, in Rotterdam, The Netherlands. Four dedicated working groups with approximately 15 allocated participants each were formed in advance. The allocation was based on specific participant characteristics: (i) personal and professional background; (ii) national diversity in each group, (iii) stakeholder diversity within each group (clinicians’ representatives, patients and patients’ representatives, regulators, HTDs, HTA bodies, payers, policy makers, and academic representatives); and (iv) participation mode (i.e., on-site vs. remote). The purpose of the four break-out sessions was to identify and prioritise a list of activities that addressed the key aspects of their respective topics:

- Topic 1—Balancing Interaction: EU HTA and EMA (WG1).
- Topic 2—Balancing Expert Input: JSC and JCA (WG2).
- Topic 3—Balancing Interface: EU HTA and MS (clinician and patient perspective) (WG3).
- Topic 4—Balancing Bias due to Stakeholder Interests: Transparency and Expertise (WG4).

### 2.3. Procedural Approach of the Break-Out Sessions

The parallel break-out sessions were held on the second day of the convention and lasted 90 min. The four sessions aimed to facilitate meaningful discussions, encourage equal participant input, and produce actionable outcomes for further elaboration in the plenary session.

### 2.4. Plenary Session and Ranking

The results of the four break-out sessions were presented in the final plenary session by each WG’s appointed representative followed by re-ranking of the top three identified action points from each WG using an online IT-based system as described previously [14,25]. The identified key topics were discussed in detail by the attendees to determine any additional points of relevance.

### 2.5. Data Handling and Analysis

Data analysis was performed as described previously [15].

The raw data supporting the conclusions of this article will be made available by the authors on request.

## 3. Results

### 3.1. The Outcomes of the Pre-Convention Survey

The pre-convention survey received 58 responses representing 13 countries/regions (global, Europe), and a wide range of stakeholder types. Key stakeholder groups included: academia (n = 6), clinicians (n = 7), HTA bodies (n = 5), health technology developers (HTD; n = 19), patient representatives (n = 7), payers (n = 5), policy makers (n = 2), and regulators (n = 7). Additionally, responses were provided by representatives having responsibilities spanning different geographical levels: global (n = 6), European (n = 13), or national (n = 39). The national representation included: the Baltic States (n = 1), Belgium (n = 3), France (n = 6), Germany (n = 8), Ireland (n = 1), Italy (n = 3), Malta (n = 1), The Netherlands (n = 6), Poland (n = 2), Spain (n = 3), Sweden (n = 1), Switzerland (n = 3), and, for non-European countries, India (n = 1).

**Figure 1:**
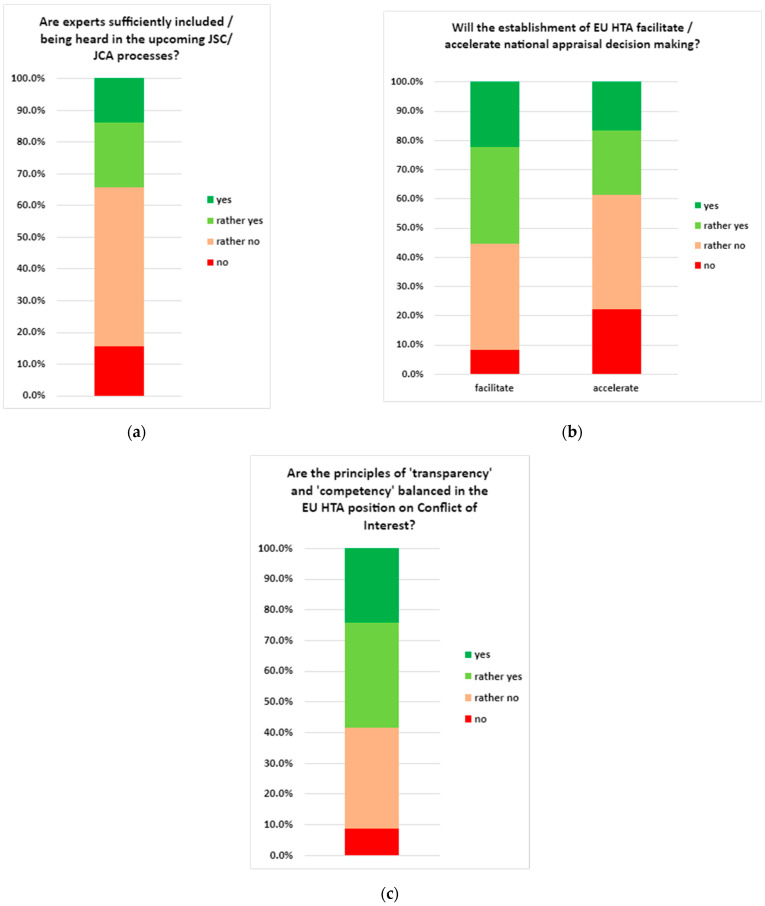
(**a**) Outcome of the pre-convention survey with the percentage of respondents (of a total of n = 58) who answered “yes”, “rather yes”, “no”, or “rather no” to the question regarding the involvement of experts in the JSC and JCA. JCA: joint clinical assessment; JSC: joint scientific consultation. (**b**) Outcomes of the pre-convention survey with the percentage of respondents (of a total of n = 58) who answered “yes”, “rather yes”, “no”, or “rather no” to the questions regarding the interface of the EU HTA and national decision-making. EU: European Union; HTA: health technology assessment. (**c**) Outcome of the pre-convention survey with the percentage of respondents (of a total of n = 58) who answered “yes”, “rather yes”, “no”, or “rather no” to the questions regarding the EU HTA position* on conflict of interest. EU: European Union; HTA: health technology assessment. * NB: the survey was conducted prior to the publication of the Draft Implementing Act on Conflict of Interest.

Figure 1 shows the outcomes of the rating questions on optimising expert input into JSC and JCA (Figure 1a), optimising the interface of the EU HTA and national HTA bodies’ methods and processes from a patient and clinician perspective (Figure 1b), and bias due to stakeholder interests (Figure 1c). Combining the answers to the rating questions into “yes”/“rather yes” vs. “no”/“rather no” resulted in the following findings:

- Optimising expert (patients, clinical experts) input into JSC and JCA: 65.5% of the respondents believe that experts are not sufficiently included/being heard in both JSC and JCA.
- Optimising the interface of the EU HTA and national HTA methods and processes: 56.9% of the respondents indicated that the EU HTA will facilitate national appraisal decision-making; however, a minority of respondents—37.9%—thought it would accelerate national appraisal decision-making.
- Bias due to stakeholder interest: 58.6% of the respondents believed that the principles of ‘transparency’ and ‘competency’ are balanced in the current (i.e., before publication of the draft IA on CoI rules under the EU HTAR by the European Commission) EU HTA position on CoI.

### 3.2. Insights from the Break-Out Sessions

**Figure 2:**
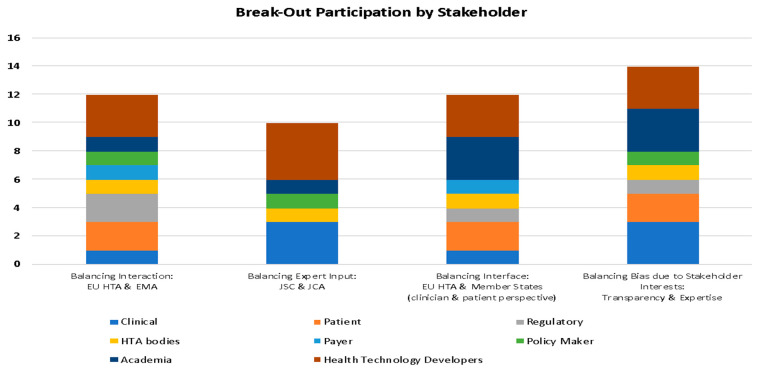
An overview of the distribution of different stakeholder profiles over the four break-out sessions. EMA: European Medicines Agency; EU: European Union; HTA: health technology assessment; JCA: joint clinical assessment; JSC: joint scientific consultation.

The numbers of participants per breakout session were, respectively: 12 for Balancing Interaction: EU HTA and EMA (WG1), 10 for Balancing Expert Input: JSC and JCA (WG2), 12 for Balancing Interface: EU HTA and MS (clinician and patient perspective) (WG3), and 14 for Balancing Bias due to Stakeholder Interests: Transparency and Expertise (WG4). While the planned allocation had included 15 participants per session (see Section 2), the reduced final numbers were due to last-minute cancellations and several no-shows. Each break-out session included representatives of a variety of national backgrounds (Belgium, France, Germany, Italy, The Netherlands, Portugal, Serbia, Spain, Sweden, Switzerland, and the UK), as well as stakeholder profiles (Figure 2). Key insights derived from each break-out session are as follows:

- WG 1: The WG reached a consensus that further collaboration on the therapeutic area methodological guidelines should be aimed for. The participants remained divided on whether the methodologies used to evaluate clinical evidence to support marketing authorisation and relative effectiveness should be further aligned or remain separate due to the differing scope of the two procedures. Further, the importance of an early scientific dialogue was highlighted. Improved communication between the EMA and EU HTA was considered necessary to ensure efficient knowledge transfer from the Committee for Medicinal Products for Human Use, especially the reasoning of the regulatory assessment, to subsequent HTA-related discussions. The WG further elaborated on potential synergies between the EMA regulatory assessment and JCA and opportunities to avoid the duplication of work. For example, the group suggested that, in case a PICO (Population, Intervention, Comparator, Outcomes) identified for a JCA has already been addressed in the marketing authorisation assessment, the JCA might refer to the regulatory assessment for HTA purposes. Table 1 depicts the three key action points identified by WG 1.
- WG 2: While the official “touch points” to include the patient and clinician voice in the EU HTA appeared well defined to the group, the details of when and how to provide this input (i.e., timelines, format, etc.) appeared less clear. The WG insisted on the importance of incorporating patients’ and clinicians’ input in the EU HTA to provide essential guidance on PICOs, insights into certain aspects of trial designs, and guidance on the evaluation of uncertainty in a specific context. The WG stressed that to leverage expertise and align efforts effectively and efficiently—considering the prevailing capacity constraints of the involved stakeholders—all experts (e.g., patients, clinicians, HTDs) should be involved early in the EU HTA process, ideally from horizon scanning and JSC. Details on when and how, and what kind of input will be collected, appeared not sufficiently clear. Furthermore, the WG considered that representativeness across different countries should be aimed for, in order to achieve comprehensive coverage and the balance of every EU region and their specific requirements and health environments. The group also pointed out that clarifying the potential impact that expert input will have at the different process stages of an EU assessment might help achieve a sustained high-quality engagement of experts. The three top priority points identified by WG 2 are detailed in Table 1.
- WG 3: There was clear consensus that, with respect to involvement in the EU HTA, the efforts of both patients and clinicians appear fragmented across the EU. This contrasts with the situation on a national level, where both act in alignment in their specific health environments. The WG considered that expert involvement in EU JCAs will likely reduce this fragmentation due to the participation and collaboration of national associations or societies both at national and the EU level. Concerns were, however, raised regarding both workload and timelines, which appear not feasible based on current guidance. Therefore, it will be important to involve clinicians and patients early and share horizon scanning results to allow for upfront discussion and preparation. To further address timelines and capacity concerns, the WG suggested clarifying processes for coordination at both the EU and national levels, and harmonising methods of expert involvement at the national level. Lastly, the WG found both political willingness and financial support important issues for increasing patient involvement capacities. The top three activities identified by WG 3 are detailed in Table 1.
- WG 4: The WG agreed that, when considering CoI in HTA, both transparency and expertise are the two equally crucial core principles. The management of CoI should be prioritising inclusion of the best available expertise while providing full transparency on relationships and interests. The group found that in the EUnetHTA 21 CoI guidance these principles was not optimally balanced, as there were concerns about the exclusion of certain groups (e.g., patient organisations co-sponsored by industry) due to CoI. The WG argued that the existence of a certain level of CoI should be accepted, but made transparent, to avoid important and valuable expertise being excluded. As a result, there should be an emphasis on the quality of the evaluation, and CoI should be managed effectively and with flexibility to meet this standard, i.e., aiming for decisions on CoI to be taken on a case-by-case basis, taking into account the individual context. The suggested three highest-priority points of relevance identified by WG 4 are stated in Table 1.

### 3.3. Ranking Obtained in the Final Plenary Session

**Figure 3:**
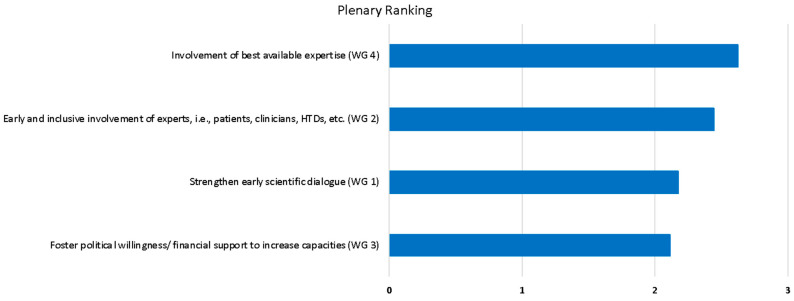
The top priorities of the break-out session insights as identified in the final plenary session, ranked by cumulative weighted responses. EMA: European Medicines Agency; EU: European Union; HTA: health technology assessment; HTD: health technology developer.

Figure 3 lists the highest priority action points of each of the four break-out sessions, as determined by ranking of the audience in the final plenary session and comparing relative weights of each item: (i) the involvement of the best available expertise (2.63 points), (ii) the early and inclusive involvement of experts, i.e., patients, clinicians, HTDs, etc. (2.45 points), (iii) the strengthening of early scientific dialogue (2.18 points), and (iv) the fostering of political willingness/financial support to increase capacities (2.12 points). Three out of top four points focused on the optimisation of the involvement of experts in EU HTA procedures and two of them related to early involvement in the EU HTA processes. Early information exchange and process guidance are seen as crucial for optimal expert input and efficient collaboration.

## 4. Discussion

The right balance in various aspects of the EU HTA/JCA is crucial. The Regulation envisions HTA to offer ‘the best outcomes for patients and society as a whole’ [2]. This entails the need for a balance between assigning an additional benefit to a health technology that does not offer the best outcomes on one side and not acknowledging an additional benefit that does exist on the other side. Appraisal decisions at the national or regional level are of significant importance, as the ramifications of the respective errors within the national access and supply context are crucial for achieving the optimal balance. Furthermore, the EU HTAR acknowledges the importance of strengthening the ‘balance in the pharmaceutical systems in the [EU] and its Member States’ [3]. An opportunity to implement balance in the EU HTA are the IAs which set the framework for key aspects of the related processes and procedures.

To approach this complex topic with its multi-faceted challenges, we utilised the established approach of analysing expert input, which is commonly used, e.g., by the European Commission [20,26]. Thereby, both quantitative and qualitative data representing the perspectives of various relevant stakeholder groups were generated [12]. We evaluated and discussed balance (i) in the interaction of the EU HTA and EMA, (ii) in input by experts for JSC and JCA, (iii) in the interface of the EU HTA and MSs from a clinician and patient perspective, and (iv) in bias due to stakeholder interest, i.e., between transparency and expertise, in light of the respective relevant IAs where a draft or final version was available [4,5,6,7].

### 4.1. Balancing Interaction for Efficient and Effective Collaboration

While the EU HTA and EMA have separate remits, the discussions of WG 1 underlined that synergies do exist, that these should be utilised, and that an early and efficient knowledge transfer should be enabled to reduce the duplication of efforts and align on topics and research questions relevant for both—the evaluation of benefits and risks and the evaluation of comparative clinical benefits. This is in line with previous work in this field by the EAA and others identifying continuous involvement in the regulatory process as playing a crucial role in facilitating an efficient and predictable JCA process for all stakeholders [11,18,27,28,29,30]. However, details on the level and specific aspects of alignment remain a topic of debate in the field. For example, at the regulatory level (both the EMA and several national levels), general methodologies used to evaluate clinical evidence have been adjusted to fit developments in specific cases (e.g., the development of targeted medicines). The consequences of these changes for the generation of clinical evidence to support regulatory assessments have not been reflected in the evidence and methodology requirements for many current HTAs, nor the EU HTA, though. Hence, one could conclude that an alignment, at least if based on HTA requirements, might represent a step back rather than step forward in achieving optimal processes [10,31,32,33]. Importantly, the EU HTAR foresees an early EU HTA dialogue that remains to be implemented after 12 January 2025.

Shortly after the EAA Convention, the *Draft IA on Cooperation between the Coordination Group and EMA* was published. This IA is solely focused on details of the exchange of information and does not cover the topic of content alignment [7]. Specifically, no guidance is given on alignment of the regulatory and EU HTA procedures in terms of requirements for outcome measures, where currently the EMA and EU HTA views differ substantially [32]. This can be regarded as a missed opportunity to address this sensitive yet essential topic; alternatively, this might be seen as a result of the different remits of the EMA and HTA. The determination to approach this challenge is apparent in the recent collaborative and future HTA—EMA workshops, that focused on evidence challenges and managing uncertainty [34].

In terms of EU—MS collaboration, the survey responses showed that, while at this stage the majority view is that JCAs will facilitate national appraisal decision-making, only a minority believe it will also accelerate national decisions. This is of high relevance for patient access to health technologies and could therefore pose a threat to a major goal of the EU HTAR [8,20,35]. As the JCAs aim to address MS requirements, which will in some cases differ due to national health system contexts, the EU HTA is an opportunity to achieve closer alignment and ultimately an EU Health Union [3,19,29,36].

At the same time, it is obvious that individual expert input will inevitably depend on their national and local environment. Contributions by patient associations and clinical and scientific societies would complement the national views and should therefore be encouraged. This would also foster interaction across the currently fragmented national patient associations and clinical societies and the development of an EU discussion [37,38]. However, early and inclusive involvement of experts, e.g., by sharing horizon scanning results to enable upfront discussions in national associations or societies, requires financial support and political willingness. As the implementation of the EU HTA joint procedures draws closer, the latter point was voted higher in the plenary ranking than the topic of the early involvement itself.

Efficient and effective collaboration with all relevant stakeholders has long been identified as a crucial element for a successful implementation of the EU HTAR [9,12,13,14,39,40]. The EAA survey and convention outcomes confirm this view with the list of identified action points and by the fact that the majority of points ranked as key issues in the final plenary session revolve around collaboration.

### 4.2. Balancing Expert Input for Efficient and Effective Inclusion

Evidence-based medicine relies on three pillars of evidence: (i) the best internal and external evidence, (ii) patient values and expectations, and (iii) clinical experience [41]. In HTA, patients’ and clinicians’ perspectives play an important role in balancing the technical scope of the assessment of best scientific external evidence by providing clinical context and additional, specific expertise [9,12,13,14]. To ensure that this expertise is included and considered in EU HTA procedures and contributes to high-quality and meaningful outcomes, both clear procedures and early interaction are needed. Continuous interaction throughout the JSC-JCA process can enable consistency and thereby improve efficiency, predictability, and, most importantly, the quality of the assessment. JSC play a crucial role for all stakeholders in this respect; however, a current lack of JSC capacities has been perceived when compared to the expected number of procedures per year [42,43].

While enabling and ensuring the inclusion of relevant expertise is essential, transparency on any potential conflict of interest is a key prerequisite for the quality and integrity of any outcome. Finding a balance between restricting expert input to that without any CoI and not excluding valuable expertise is a demanding task in any system and has been discussed and debated in the context of (EU) HTA at length [9,12,14,18,44]. Further, and in line with the various goals of the EU HTAR, the EMA has guidelines and documentation on CoI which largely overlap with that detailed in the EU HTAR [45,46]. Therefore, to avoid the duplication of efforts, an alignment of CoI management between the EMA and EU HTA would be desirable.

Both in the survey responses and in the discussions of WG 4, the EU HTA position on CoI was found to be on the right path to achieving a balance between transparency and competency. The draft IA on CoI was made available for public consultation after the EAA Convention and therefore was not considered by the working group [6]. In the draft IA on CoI, an approach excluding any CoI with relation to HTD interaction (but not other types of CoI) is prioritised over balancing the need for valuable expertise with transparency on bias. Despite this topic being highly specific, more than 80 individual feedback statements were submitted to the Commission [47]. It remains to be seen how the submitted feedback will be considered and implemented in the final IA, and how the rules set out by the IA will be interpreted and applied in the upcoming procedures from January 2025.

### 4.3. Limitations and Further Research

While our research provides timely and relevant insights into various key topics for the implementation of the EU HTAR, the scope of this research might still be considered exploratory.

Utilising the approach of expert input for research is an established and powerful method, albeit not without certain limitations. Potential bias due to personal views and/or the over-representation of the perspectives of single leading experts needs to be considered [20,26,48,49,50]. To address this potential limitation, we aimed to generate input, both in the survey and the discussions at the convention, from a wide variety of experts with the relevant expertise and varying backgrounds and avoided overcomplexity in the number of questions to be addressed. In addition, discussions during the convention were moderated by independent break-out leads to ensure input by all participants and avoid single perspectives dominating the discussion.

The representation of stakeholders, geographical and national differences, and individual perspectives is limited despite our efforts to ensure a broad representation of stakeholder groups and national backgrounds both for the survey and for the convention participants. Nevertheless, the sample of respondents and participants is considered representative and allows for conclusions to be drawn. Invitations to participate were distributed in a multi-channel approach utilising online (e.g., via the EAA website, email, professional social media, online platforms, and expert networks), as well as conventional methods (e.g., via direct communication) to account for varying use or reach of these channels. In addition, participation in the convention was possible on-site and remotely to account for varying distances, the ability to travel or the availability of travel options, etc. Participation in the WG at the convention was limited to no more than four representatives of one stakeholder type in each group, leading to rather balanced WG compositions (Figure 3). The number of participants per group [10,11,12,13,14] and the methodology for collecting questions during discussion sessions (on-site by hand-sign as well as virtually) allowed all participants to actively contribute, minimising bias through participation-mode or background. Geographical representation covered a variety of European countries and their unique national experiences within each stakeholder group, both in the survey and the convention. Overall, there was still a broad range of perspectives represented and hence diverse viewpoints were included in the survey and convention outcomes.

In order to address the limitations as discussed above, future research might, for instance, analyse feedback from a larger sample of respondents, ensure the equal representation of stakeholder representatives and national backgrounds, and/or exclude or separately analyse multi-national representatives.

Some relevant IA were only published by the European Commission after the responses to the pre-convention survey were collected and after the EAA Convention took place. While these were considered in the Section 4 of the manuscript, future research might provide additional insights in the context of the final rules for the implementation of the EU HTAR.

Further research on the key topics for balance in the implementation of the EU HTA will be conducted by the EAA during the Fall Convention 2024, where early and inclusive involvement of experts will be employed in working group discussions regarding challenges with multiple PICOs and indirect treatment comparisons. The involvement of and activities by EU MSs will be elaborated in depth utilising the example of Italy, with relevant participants in the respective working group at the convention in Rome. The outcomes of this work will be published elsewhere.

## 5. Conclusions

The most challenging task of the EU HTA is to find the right balance between being too “lenient” and being too “strict” in the evolving HTA process and in the approach to comparative evidence. Defining and achieving the right balance is particularly important when the goal is to improve patient access to life-saving innovative health technologies. This becomes challenging as MS health systems vary in their capacities to implement the JCA outcomes and make these treatments available to patients. Four dimensions of ‘finding the right balance’ were explored in more detail during the EAA Spring Convention 2024. Optimising the implementation of the EU HTA requires an approach to conflict of interest that balances transparency obligations and the need for relevant and meaningful expertise, strengthens the involvement of clinical and patient experts, intensifies early interaction between the EMA and EU HTA and increases the involvement and activity of the EU MSs. With the first joint procedures beginning in January 2025, there will be a steep learning curve regarding implementing the framework as outlined in the regulation and IAs, as well as regarding establishing clear criteria for measuring success.

## Figures and Tables

**Table 1:** Overview and ranking of the top three key insights generated during each break-out session *.

Priority	Balancing Interaction: EU HTA and EMA	Balancing Expert Input: JSC and JCA	Balancing Interface: EU HTA and MS (Clinician and Patient Perspective)	Balancing Bias Due to Stakeholder Interests: Transparency and Expertise
1	Strengthen early scientific dialogue	Early and inclusive involvement of experts, i.e., patients, clinicians, HTDs, etc.	Share results of horizon scanning with MS and facilitate upfront discussion on PICOs both by patients and clinicians and national and EU level (scientific advice)	Involvement of best available expertise
2	(Better) Communicate CHMP reasoning applicable for subsequent HTA discussion	Aim for balanced representation of countries	Foster political willingness/financial support to increase capacities	Ensure full transparency
3	Collaborate on therapeutic area related methodological guidelines	Clarify official touchpoints (timelines, format)	Install clear process on coordination across EU and national level both for clinicians and patients	Accept that CoI is there and manage it effectively and with flexibility

CHMP: Committee for Medicinal Products for Human Use; CoI: conflict of interest; EMA: European Medicines Agency; EU: European Union; HTA: health technology assessment; HTD: health technology developer; JCA: joint clinical assessment; JSC: joint scientific consultation; MS: Member States; PICOs: Population, Intervention, Comparator, Outcomes; * Wording adapted from break-out reports for grammatic consistency and readability.

## Data Availability

The raw data supporting the conclusions of this article will be made available by the authors on request.

## Supplementary Materials

The following supporting information can be downloaded at https://www.mdpi.com/article/10.3390/jmahp13010006/s1: Supplementary Material S1: EAA pre-convention survey as shared with the respondents.

## Statements and Declarations

The content of the manuscript and the conclusions drawn are derived from the discussions during the convention and may not necessarily reflect the positions of the organisations that employ the authors. All authors have read and agreed to the published version of the manuscript.
