# Supplementary material for: Avoiding Error and Finding the Right Balance in European Health Technology Assessments: Insights Generated by the European Access Academy
Source: J Mark Access Health Policy. 2025 Feb 10;13(1):6. doi: 10.3390/jmahp13010006 (PMC11843953; doi:10.3390/jmahp13010006)
Supplement: Supplementary file 1 [file jmahp-13-00006-s001.zip › jmahp-3217339-supplementary.pdf]

Questionnaire - Background Information

1

What country or region do you represent?

2

Which stakeholder group do you represent? *

- [ ] Patient representative
- [ ] Clinician / Health Care Provider / Medical Association
- [ ] Regulatory
- [ ] HTA body
- [ ] Payer
- [ ] Health Technology Developer (HTD) / HTD Associations
- [ ] Other

Balancing Interaction of EU HTA & EMA

see below for some relevant wording from the Regulation

3. In the preparation or amendment of the annual work programme, the Coordination Group shall:

(a) take into account the reports on emerging health technologies referred to in Article 22;

(b) take into account the information from the European Medicines Agency that is provided by the Commission pursuant to Article 28 on the status of submitted and upcoming marketing authorisation applications for medicinal products referred to in Article 7; as new regulatory data becomes available, the Commission shall share such information with the Coordination Group so that the annual work programme can be amended;

Article 15

Adoption of detailed procedural rules for joint clinical assessments

1. The Commission shall adopt, by means of implementing acts, detailed procedural rules for:

(a) cooperation, in particular by exchange of information, with the European Medicines Agency on the preparation and update of joint clinical assessments of medicinal products;

Article 16

Principles of joint scientific consultations

5. Joint scientific consultations on medicinal products may take place in parallel with the scientific advice from the European Medicines Agency pursuant to Article 57(1), point (n), of Regulation (EC) No 726/2004. Such parallel consultations shall involve the exchange of information and have synchronised timing, while preserving the separation of the respective remits of the Coordination Group and the European Medicines Agency. Joint scientific consultations on medical devices may take place in parallel with the consultation of the expert panels pursuant to Article 61(2) of Regulation (EU) 2017/745.

3

Which areas of interaction between EU HTA and EMA warrant further definition?
(please rank the options - highest priority on top of the list, lowest priority on the bottom) *

- Exchange of information during parallel Joint Scientific Consultation (JSC)
- Cooperation to support timely preparation of Joint Clinical Assessment (JCA) by HTA
- Initiation of HTA dossier assessment in relation to EMA procedure
- Content interaction on evidence planning (applicable study design, patient-relevance of endpoints, appropriate comparative therapy)
- Sharing information relevant for lead Population/ Intervention/ Comparator/ Outcome/ Study Design (PICOs) scheme(s)
- Management of changes to the therapeutic indication during the regulatory process
- any other (please specify below)

4

If there are any other areas of interaction between EMA & HTA warranting further scrutiny please specify them here:

Optimizing Expert (patients, clinical experts) Input into JSC & JCA

see below for some relevant wording from the regulation

(44) In order to ensure the inclusiveness and transparency of the joint work, the Coordination Group should engage and consult widely with stakeholder organisations with an interest in Union cooperation on HTA, including patient organisations, healthcare professional organisations, clinical and learned societies, health technology developer associations, consumer organisations and other relevant non-governmental organisations in the field of health. A stakeholder network should be set up to facilitate dialogue between stakeholder organisations and the Coordination Group.

(45) In order to ensure that joint work is of the highest scientific quality and reflects the state of the art, external experts with relevant in-depth specialised expertise should provide input on joint clinical assessments and joint scientific consultations. Such experts should include clinical experts in the therapeutic area concerned, patients affected by the disease, and other relevant experts on, for example, the type of health technology concerned or issues related to clinical study design. European Reference Networks could also be used as source to identify those experts and access

L 458/8 EN Official Journal of the European Union 22.12.2021

relevant knowledge in specific therapeutic areas. Patients, clinical experts and other relevant experts should be selected for their subject matter expertise and act in individual capacity rather than representing any particular organisation, institution or Member State. In order to preserve the scientific integrity of the joint clinical assessments and joint scientific consultations, rules should be developed to ensure the independence and impartiality of patients, clinical experts and other relevant experts involved, and avoid conflicts of interest.

5

Are experts sufficiently included / being heard in the upcoming JSC/ JCA processes?
(1 star: no; 4 stars: yes) *

☆ ☆ ☆ ☆

6

What areas of expert involvement warrant further scrutiny?
(please rank the options - highest priority on top of the list, lowest priority on the bottom) *

- Early involvement of experts in the JSC
- Early involvement of experts in the JCA
- Having an opportunity for experts to comment on the draft JSC outcome
- Having an opportunity for experts to comment on the draft JCA report
- Having an opportunity for experts to comment on the draft PICOs scheme(s)
- Collaboration between national and European experts/ expert organisations
- If experts should act in individual capacity rather than representing any particular organisation, institution or Member State
- any other (please specify below)

7

If there are any other areas of expert involvement warranting further scrutiny please specify them here:

Optimizing the Interface of EU HTA and National HTA Methods and Processes

see below for some relevant wording from the regulation

(9) The European Parliament, in its resolution of 2 March 2017 on EU options for improving access to medicines ([7]), called on the Commission to propose legislation on a European system for HTA as soon as possible and to harmonise transparent HTA criteria in order to assess the added therapeutic value and relative effectiveness of health technologies compared with the best available alternative, that takes into account the level of innovation and benefit for patients.

(36) The timeframe for joint clinical assessments for medicinal products should be fixed, as far as possible, by reference to the timeframe applicable to the completion of the centralised marketing authorisation procedure provided for under Regulation (EC) No 726/2004. Such coordination should ensure that joint clinical assessments could effectively facilitate market access and contribute to the timely availability of innovative health technologies for patients. Health technology developers should therefore respect the deadlines established pursuant to this Regulation when submitting the requested information, data, analyses and other evidence.

(54) In order to ensure that the support framework continues to be as efficient and cost-effective as possible, the Commission should report to the European Parliament and to the Council on the application of this Regulation no later than three years after its date of application. The report should focus on reviewing the added value of the joint work for the Member States. In particular, the report should consider whether there is a need to introduce a fee-paying mechanism, which would ensure the independence of the Coordination Group, through which health technology developers would also contribute to the financing of joint scientific consultations. In addition, the report should review the effect of the non-duplication of the request of information, data, analyses and other evidence for joint clinical assessment in terms of reducing the administrative burden for the Member States and health technology developers, facilitating market access for new and innovative products and reducing costs. The report could trigger an assessment on the progress made regarding patient access to innovative health technologies, the sustainability of health systems and the HTA capacity at Member State level.

8

Will the establishment of EU HTA facilitate national appraisal decision making ?
(1 star: no; 4 stars: yes) *

☆ ☆ ☆ ☆

9

Will the establishment of EU HTA accelerate national appraisal decision making ?
(1 star: no; 4 stars: yes) *

☆ ☆ ☆ ☆

10

What areas of interaction warrant further scrutiny?
(please rank the options - highest priority on top of the list, lowest priority on the bottom) *

- Timelines
- Pending adjustments of national HTA procedures and requirements
- Details of supplementary national evidence requirements
- Alignment on Population/ Intervention/ Comparator/ Outcome/ Study Design (PICOS) scheme(s)
- Unequal requirements for drugs assessed within the first/ second/ third wave of the implementation of the EU HTA Regulation
- any other (please specify below)

11

If there are any other areas of interaction warranting further scrutiny please specify them here:

Bias due to Stakeholder Interests

see below for some relevant wording from the regulation

Article 5

Transparency and conflict of interest

1. The Coordination Group shall carry out its activities in an independent, impartial and transparent manner.

2. The representatives appointed to the Coordination Group and its subgroups, and patients, clinical experts and other relevant experts participating in any joint work, shall not have any financial or other interests in the health technology developers' industrial sector which could affect their independence or impartiality.

3. The representatives appointed to the Coordination Group and its subgroups shall make a declaration of their financial and other interests and update it annually and whenever necessary. They shall disclose any other facts of which they become aware that might in good faith reasonably be expected to involve, or give rise to, a conflict of interest.

4. The representatives who participate in meetings of the Coordination Group and its subgroups shall declare, before each meeting, any interest which could be considered to be prejudicial to their independence or impartiality with respect to the items on the agenda. Where the Commission decides that a declared interest constitutes a conflict of interest, that representative shall not take part in any discussions or decision-making, or obtain any information concerning that item of the agenda. Such declarations of representatives and the decision of the Commission shall be recorded in the summary minutes of the meeting.

5. Patients, clinical experts and other relevant experts shall declare any financial and other interests relevant to the joint work in which they are due to participate. Such declarations and any actions taken as a result shall be recorded in the summary minutes of the meeting and in the outcome documents of the joint work in question.

6. The representatives appointed to the Coordination Group and its subgroups as well as patients, clinical experts and other relevant experts involved in the work of any subgroup shall, even after their duties have ceased, be subject to a requirement of professional secrecy.

12

Are the principles of 'transparency' and 'competency' balanced in the EU HTA position on Conflict of Interest (CoI)?
(1 star: not balanced; 4 stars: optimally balanced) *

☆ ☆ ☆ ☆

13

What areas of bias due to stakeholder interests warrant further scrutiny?
(please rank the options - highest priority on top of the list, lowest priority on the bottom) *

- Definition of CoI (what constitutes a CoI?)
- Applicability of EMA's CoI management system in order to avoid duplication of effort
- Interface of learned societies and individual experts to overcome CoI
- any other (please specify below)

14

If there are any other areas of bias warranting further scrutiny please specify them here:

Add-On Question: Assessment of Uncertainty in Evidence

Background:
HTA is based on the concept of Evidence Based Medicine (EBM) with the three pillars of the EBM Triade being i) Best Internal and External Evidence; ii) Patient Values and Expectations; and iii) Clinical Experience. When the first pillar (i.e., evidence) is weaker due to increased levels of uncertainty, pillars two (i.e., patient perspectives) and three (clinical experience) will need to be strengthened to regain balance [1].

[1] Sackett DL, Rosenberg WM, Gray JA, Haynes RB, Richardson WS. Evidence based medicine: what it is and what it isn't. BMJ. 1996 Jan 13;312(7023):71-2. doi: 10.1136/bmj.312.7023.71.

see below for some relevant wording from the regulation

(3) HTA is able to contribute to the promotion of innovation, which offers the best outcomes for patients and society as a whole, and is an important tool for ensuring proper application and use of health technologies.

Article 9

Joint clinical assessment reports and the dossier of the health technology developer

1. A joint clinical assessment shall result in a joint clinical assessment report that shall be accompanied by a summary report. Those reports shall not contain any value judgement or conclusions on the overall clinical added value of the assessed health technology and shall be limited to a description of the scientific analysis:

(a) of the relative effects of the health technology as assessed on the health outcomes against the chosen parameters which are based on the assessment scope as set out pursuant to Article 8(6);

(b) of the degree of certainty of the relative effects, taking into account the strengths and limitations of the available evidence.

15

Is EU HTA sufficiently prepared to face expected uncertainty in evidence to be provided in the EU HTA submissions?
(1 star: no; 4 stars: yes) *

☆ ☆ ☆ ☆

16

What are suitable approaches to address residual questions and remaining uncertainty regarding evidence in order to increase confidence in decision making?
(please rank the options - highest priority on top of the list, lowest priority on the bottom) *

- Integration of the clinical perspective into the assessment
- Integration of the patient perspective into the assessment
- Integration of HTD expertise throughout the process
- Balancing established and innovative methods for comparative assessments
- any other (please specify below)

17

If there are any other suitable approaches to address residual questions and remaining uncertainty regarding evidence in order to increase confidence in decision making please specify them here:
